# The first ethanol sclerotherapy of an accessory cavitated uterine mass

**DOI:** 10.1002/ccr3.3371

**Published:** 2020-11-29

**Authors:** Philippe Merviel, Caroline Lelievre, Tatiana Cambier, Isabelle Thomas‐Kergastel, Pierre‐François Dupré

**Affiliations:** ^1^ Gynecology and Obstetrics Department Brest University Hospital Brest France; ^2^ Radiology Department Brest University Hospital Brest France

**Keywords:** accessory cavitated uterine mass, dysmenorrhea, ethanol, sclerotherapy, uterine rupture

## Abstract

Accessory cavitated uterine mass (ACUM) is a very rare, underdiagnosed pathology. It is treated with radical surgery, which results in uterine scarring. Here, we describe the first case of ethanol sclerotherapy of an ACUM, modeled on the treatment of recurring endometrioma. Ethanol sclerotherapy avoids uterine scarring and the secondary risk of uterine rupture and enables the rapid achievement of pregnancy.

## Graphical abstract

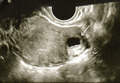

## 1 INTRODUCTION

Accessory cavitated uterine mass (ACUM) is a very rare, underdiagnosed pathology. It is treated with radical surgery. Here, we describe the first case of ethanol sclerotherapy of an ACUM. Ethanol sclerotherapy avoids uterine scarring and the secondary risk of uterine rupture and enables the rapid achievement of pregnancy.

An accessory cavitated uterine mass (ACUM) was described for the first time in 1996 by Tamura et al as a juvenile adenomyotic cyst.^1^ In 2010, Acien et al ^2^ suggested that a noncommunicating uterine mass containing functional endometrium could be referred to as an ACUM. The mass is responsible for severe, recurrent dysmenorrhea and pelvic pain. It prompts frequent consultations and then laparotomic or coelioscopic excision of the tumor, leading to uterine scarring. By analogy with the treatment of endometrial cysts, we developed ethanol sclerotherapy as a conservative treatment for ACUM.

## 2 CASE REPORT

**Figure 1:**
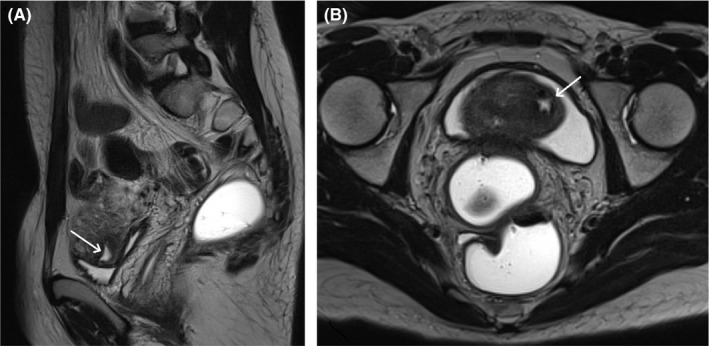
Magnetic resonance imaging: A, a para‐axial view of the left lateral boundary of the uterus (T2 sequence). The white arrow indicates the ACUM (a hyperdense cavity). B, a cross‐sectional view (T2 sequence). The white arrow indicates the ACUM (a hyperdense cavity)

**Figure 2:**
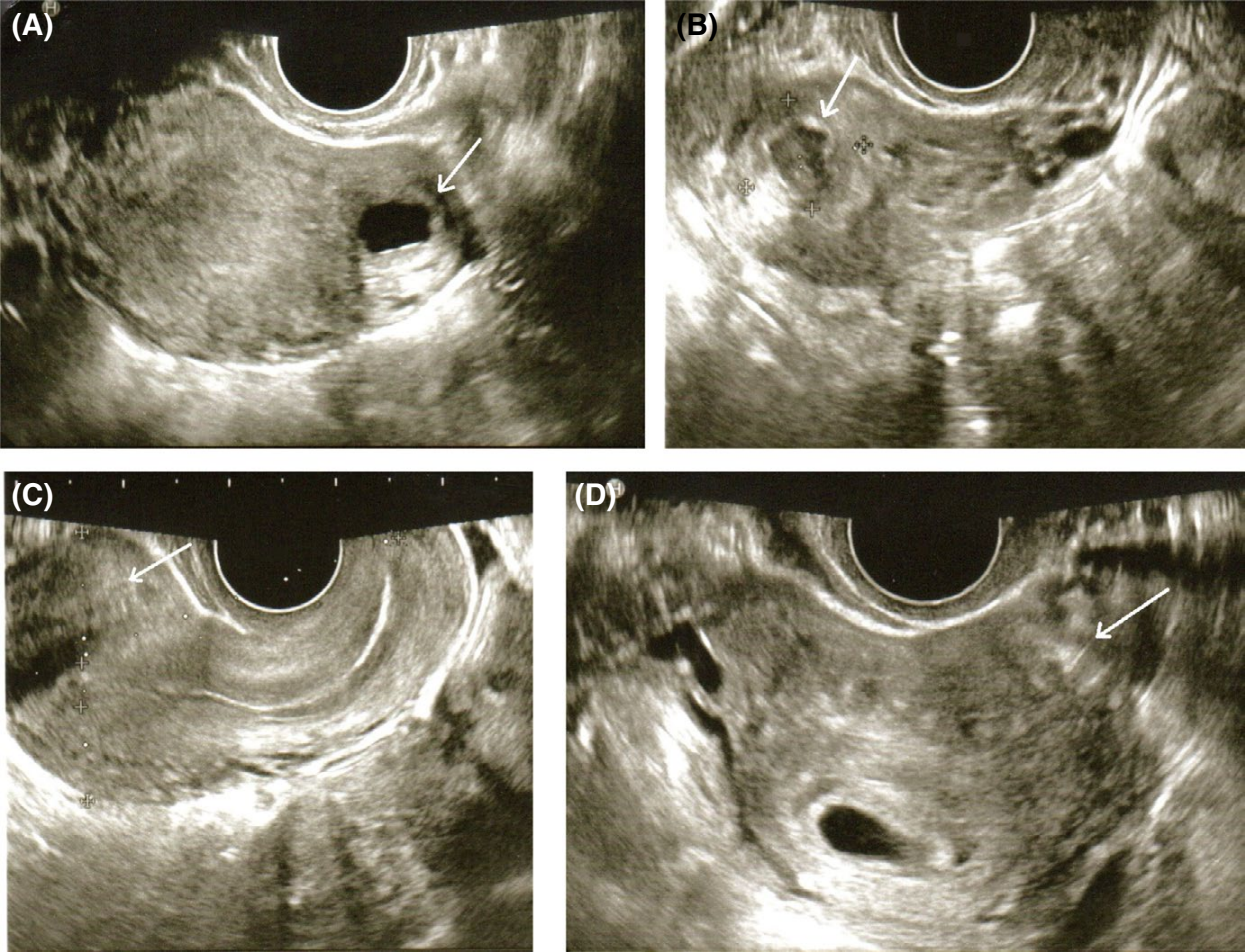
Vaginal pelvic ultrasound: A, a pre‐sclerotherapy cross‐sectional view of the ACUM near the left uterine horn. The white arrow indicates the ACUM or its former site. B, a parasagittal view during sclerotherapy of the ACUM, after ethanol injection. The white arrow indicates the ACUM or its former site. C, a post‐sclerotherapy parasagittal view at 1 mo (disappearance of the ACUM). The white arrow indicates the ACUM or its former site. D, a cross‐sectional view at the start of pregnancy (6 wk of amenorrhea), 3 mo after sclerotherapy (disappearance of the ACUM). The white arrow indicates the ACUM or its former site

A 27‐year‐old woman consulted for an ACUM in the left uterine horn. Her medical history included hysteroscopy‐coelioscopy at the age of 19 for pelvic pain refractory to treatment with step 3 analgesics and suspected noncommunicating left hemi‐uterus with hematometra on MRI. The hysteroscopy showed that the uterine cavity was normal, with no malformations. Coelioscopy revealed a nodule in the left anterolateral uterine fundus. The tumor was then drained (via a direct, 1 cm incision) but not excised. The release of a chocolate‐brown liquid appeared to confirm the diagnosis of cystic adenomyosis. The incision was sutured with a cross‐stitch pattern. At the age of 23, the patient became pregnant. A male newborn (birthweight: 3500 g) was delivered by caesarean section in an indication of mechanical dystocia. Two years later, persistent, repeated, cramp‐like pelvic pain prompted the women to consult. Magnetic resonance imaging revealed left lateral fundal adenomyoma (outer diameter: 28 mm; lumen diameter: 10 mm) but no associated adenomyosis (Figure 1A and 1B). Several treatments were prescribed (a microdose progestogen contraceptive, a norpregnane progestogen contraceptive, a levonorgestrel intrauterine device, dienogest, and step 2 and 3 analgesics) but did not relieve the patient's symptoms. At the age of 27, the woman consulted again because she wished to become pregnant again, despite the recrudescence of very focal pain in the left uterine horn. Pelvic ultrasound revealed a mass (diameter: 28 mm; lumen: 10 mm) in the left uterine horn, some distance from the uterine cavity (Figure 2A). A diagnostic hysteroscopy showed that the uterine cavity was normal and did not communicate with the mass. With the patient's consent, we decided to perform ethanol sclerotherapy in order to (a) avoid another coelioscopy and tumor excision and (b) enable pregnancy more rapidly. In the operating room and with vaginal ultrasound guidance, we extracted 5 mL of chocolate‐brown liquid and then placed 5 mL of 96% alcohol in the cavity for 15 minutes (Figure 2B). At the end of the operation, we recovered all the alcohol. The woman was discharged to home two hours later. One month after the operation, the woman was asymptomatic. Pelvic ultrasound showed that the ACUM had not recurred (Figure 2C). Two months after ethanol sclerotherapy, the woman became pregnant. The pelvic ultrasound results for the left uterine horn were normal (Figure 2D). During the subsequent caesarean delivery (indicated for prolonged pregnancy, labor failure, and uterine scarring), no nodule in the left uterine horn was noted. At a follow‐up consultation with the patient at the age of 29, a pelvic ultrasound assessment showed that the ACUM had not recurred.

## 3 DISCUSSION

For a diagnosis of ACUM, several criteria must be met: an isolated cyst‐like mass, a normal uterine cavity, normal fallopian tubes and ovaries, exeresis of the mass with a pathology assessment, an accessory cavity bordered by endometrial epithelium composed of glands and stroma, the presence of chocolate‐brown liquid in the cyst, and the absence of adenomyosis (if hysterectomy has been performed).^2^ According to Acien et al, an ACUM is usually located near the insertion of the left or right round ligament and may correspond to dysfunction of the gubernaculum.^3^ In fact, the ACUM might be caused by ectopia or by the duplication or persistence of ductal Müllerien tissue close to where the round ligament is attached. The ACUM is categorized as U6 (“still unclassified”) in the ESHRE/ESGE classification.^4^ This clinical entity is most frequently described in nulliparous women under the age of 30 who experience pelvic pain throughout the menstrual cycle.^5^ Ultrasound reveals a mass with a central cavity situated in the wall of the uterine horn. Furthermore, the endometrium and myometrium are normal. Magnetic resonance imaging shows T1 hyperintensity in the ACUM cavity; the hyperintensity is still present with a T1 fat‐sat sequence but is attenuated in a T2 sequence, indicating hematometra. The uterus is normal and free of malformations; contrast enhancement does not provide additional diagnostic information. ACUM‐associated renal anomalies have never been described.^3^, ^6^ It is advisable to check (using hysterosalpingography or hysteroscopy) that the mass does not communicate with the uterine cavity. The main differential diagnoses are adenomyoma, cystic adenomyosis, and myoma with necrobiosis—pathologies that are rarely isolated or located in the uterus and that mainly occur in older women. Another differential diagnosis is a noncommunicating rudimentary left uterine horn (U4a in the ESHRE/ESGE classification ^4^); however, in that instance, one observes a contralateral hemi‐uterus, which is not the case for an ACUM. Various pharmacological treatments have been suggested (oral contraceptives and GnRH agonists) but they do not fully relieve the pain, which recurs rapidly upon discontinuation of the medication.^7^ Treatment is generally based on coelioscopy (robot‐assisted or not) or laparotomy, in order to enucleate the tumor. Peripheral dissection of an ACUM is less complicated than that of an adenomyoma, due to the well‐delimited, concentric muscle fibers surrounding the ACUM’s cavity.^5^, ^8^

Here, we described the first case of ethanol sclerotherapy in an indication of ACUM; this treatment relieved the clinical symptoms (pelvic pain), enabled pregnancy rapidly, and mitigated the risk of uterine rupture. Sclerotherapy is a well‐proven treatment for endometrioma; it induces inflammation, destruction, and fibrosis of the cyst wall and, potentially, obliteration of the whole cyst.^9^ Several substances have been used: 95%‐100% ethanol, methotrexate, and tetracycline. We used 96% ethanol. In cases of endometrioma, Cohen et al ^10^ reported that success factors include the duration of ethanol instillation, the instilled volume, and retention in situ. In fact, the endometrioma relapse rate after sclerotherapy ranges from 0% to 62% when the ethanol is recovered and from 0% to 13% when it is left in situ. The relapse rate also varies with the duration of instillation (62% if the ethanol is left in place for less than 10 minutes vs. 9% for 10 minutes or more).^11^ In the case described here, we injected 100% of the aspirated volume (in general, we inject 80% of the volume in an indication of recurrent endometrioma, so that the surrounding ovarian tissue is not damaged), left it for 15 minutes, and then recovered it so as to avoid alcohol intoxication via diffusion into the circulation. In the present case, ultrasound assessments one month after sclerotherapy, during each trimester of pregnancy, and then 2 years after sclerotherapy showed that the ACUM had not recurred.

The incidence of uterine rupture after adenomyomectomy is very low (1%) ^12^; 24 cases were reported between 2004 and 2015. This frequency is the same as that reported by Dubuisson et al for uterine rupture after coelioscopic myomectomy.^13^ In the latter series, all types of myoma (subserosal and intramural) were associated with a risk of uterine rupture, regardless of the size (20‐50 mm), site, and time of occurrence (after between 17 and 35 weeks of amenorrhea, ie, mainly at the end of the second trimester or the start of the third). With regard to adenomyomectomy, several factors appear to contribute to the risk of uterine rupture: the operating technique (scissors vs electrocautery), the extent of exeresis (complete vs. partial), the exeresis volume, the method used to suture the uterine cavity and the wall of the myometrium, the incidence of postoperative infections and/or hematoma of the exeresis site, and the time interval between surgery and pregnancy onset (ie, postoperative contraception). Whether the adenomyomectomy is laparotomic or coelioscopic does not appear to influence the risk of uterine rupture,^12^ although a literature review ^14^ found that the incidence was slightly higher after coelioscopic myomectomy (0.99%) than after laparotomic myomectomy (0.66%). High‐quality suturing of the exeresis site in several planes is a key determinant of the risk of subsequent uterine rupture because it influences the absence of dehiscence or secondary hematoma, both of which may impact wound healing. It is sometimes easier to correctly suture the myometrium in the deep uterine wall by laparotomy than by coelioscopy, although this also depends on the surgeon's dexterity. The ACUM’s particular location in the uterine horn (with a thin myometrium, close to the uterine cavity and the fallopian tube) means that the suturing must be particularly precise. Cases of catastrophic uterine rupture have been described after exeresis of the uterine horn in an indication of extra‐uterine pregnancy. This is why the in situ injection of methotrexate is recommended for this type of cornual extrauterine pregnancy. The hemorrhagic risk associated with this injection (due to the local neovascularization during pregnancy) does not appear to affect transmyometrial puncture outside pregnancy, as observed for IVF oocyte retrievals when the ovary is behind the uterus.

After adenomyomectomy, the patient should wait for least 6‐12 months before trying for a pregnancy. After a caesarean section (which increases the risk of uterine rupture two‐ or three‐fold), patients used to be advised to wait 24 months before envisaging a pregnancy,^15^ although this time interval has now been shortened to 6 months.^16^ Whereas a caesarean section scar is located on the lower segment and the operating technique has been frequently improved, post‐adenomyomectomy scars are different because they are situated fully in the myometrium, are thicker, and are more hemorrhagic. Ultrasound and/or MRI investigation shows resorption of the postoperative hematoma at 6 months in 81% of cases, although this depends on the size of the adenomyoma.^17^ Hence, ethanol sclerotherapy advantageously enables pregnancy soon after surgery, as was the case here (2 months).

## 4 CONCLUSIONS

For a number of reasons (the absence of laparotomy/coelioscopy, per‐operatory bleeding and uterine scarring, the short time interval between surgery and planning a pregnancy, and the long‐term effectiveness), the physician can legitimately consider ethanol sclerotherapy for a symptomatic ACUM.

## CONFLICT OF INTEREST

None declared.

## AUTHOR CONTRIBUTIONS

PM: substantially contributed to the conception, design of the work, the acquisition, analysis, and interpretation of clinical and ultrasound data and have drafted the work or substantively revised it. CL: involved in the acquisition of clinical data. TC: involved in the acquisition of clinical data. IT‐K: involved in the acquisition of data on MRI. P‐FD: substantially contributed to the conception, the acquisition, and analysis and interpretation of clinical data.

## ETHICAL APPROVAL

The consent of the woman is available in the medical record and with the corresponding author.
